# Choroidal Thickness in Different Types of Inherited Retinal Dystrophies

**DOI:** 10.18502/jovr.v15i3.7454

**Published:** 2020-07-29

**Authors:** Hamideh Sabbaghi, Hamid Ahmadieh, Jalil Jalili, Nazanin Behnaz, Maryam Fakhri, Fatemeh Suri, Bahareh Kheiri, Mojtaba Rajabpour, Morteza Entezari, Narsis Daftarian

**Affiliations:** ^1^Ophthalmic Epidemiology Research Center, Research Institute for Ophthalmology and Vision Science, Shahid Beheshti University of Medical Sciences, Tehran, Iran; ^2^Department of Optometry, School of Rehabilitation, Shahid Beheshti University of Medical Sciences, Tehran, Iran; ^3^Ophthalmic Research Center, Research Institute for Ophthalmology and Vision Science, Shahid Beheshti University of Medical Sciences, Tehran, Iran; ^4^Medical Physics and Biomedical Engineering Department, School of Medicine, Tehran University of Medical Sciences, Tehran, Iran; ^5^Ocular Tissue Engineering Research Center, Shahid Beheshti University of Medical Sciences, Tehran, Iran

**Keywords:** Choroidal Thickness, Cone-rod Dystrophy, Enhanced Depth Optical Coherence Tomography, Inherited Retinal Dystrophy, Retinitis Pigmentosa, Stargardt Disease, Usher Syndrome

## Abstract

**Purpose:**

To compare the choroidal thickness among eyes with retinitis pigmentosa (RP), Stargardt disease, Usher syndrome, cone-rod dystrophy, and healthy eyes of sex- and age-matched individuals.

**Methods:**

In this comparative study, 503 eyes with RP (*n* = 264), cone-rod dystrophy (*n *= 109), Stargardt disease (*n* = 76), and Usher syndrome (*n* = 54) were included. To validate the data, 109 healthy eyes of 56 sex- and age-matched individuals were studied as controls. Choroidal imaging was performed using enhanced depth imaging-optical coherence tomography. Choroidal thickness was measured manually using MATLAB software at 13 points in nasal and temporal directions from the foveal center with the interval of 500 µm and the choroidal area encompassing the measured points was calculated automatically.

**Results:**

The mean age was 36.33 ± 13.07 years (range, 5 to 72 years). The mean choroidal thickness at 13 points of the control eyes was statistically significantly higher than that in eyes with RP (*P* < 0.001) and Usher syndrome (*P* < 0.05), but not significantly different from that in eyes with Stargardt disease and cone-rod dystrophy. Among different inherited retinal dystrophies (IRDs), the choroidal thickness was the lowest in eyes with RP (*P* < 0.001). Choroidal thickness in the subfoveal area correlated negatively with best-corrected visual acuity (*r* = -0.264, *P* < 0.001) and the duration of ocular symptoms (*r* = -0.341, *P* < 0.001) in all studied IRDs. No significant correlation was observed between the subfoveal choroidal thickness and central macular thickness (*r* = -0.24, *P *= 0.576).

**Conclusion:**

Choroidal thinning in four different types of IRDs does not follow a similar pattern and depends on the type of IRD and the duration of ocular symptoms. A larger cohort is required to verify these findings

##  INTRODUCTION

Photoreceptors and retinal pigment epithelial (RPE) cells as their protectors are the primary units for light photon translation into neural electric codes.^[1]^ Choroid is a complex vascular tissue that supplies oxygen and nutrients to these cells with a high metabolism rate. Therefore, a healthy choroidal vasculature could provide optimum blood flow to RPE and photoreceptor cells.^[2]^ Inherited retinal dystrophies (IRDs) are ocular diseases that primarily involve progressive degeneration of RPE and/or photoreceptor cells.^[3]^ Retinitis pigmentosa (RP) is the most prevalent type of IRD that has been estimated to affect approximately 1.5 million individuals worldwide.^[4]^ Stargardt disease is the most prevalent inherited macular dystrophy with an estimated prevalence of 1 per 10,000 individuals.^[5]^ A recent study showed that mutations in more than 120 causative genes are responsible for different types of IRDs^[6]^ and these mutations can be transmitted to the next generation by different Mendelian patterns of inheritance.^[7]^ Generally, it appears that the outer retina and RPE cells are primarily involved, resulting in death of these cells.^[8,9]^ Choriocapillaris may also be involved in the late stages of the disease, manifesting as chorioretinal atrophy in fundus examination.^[9,10]^

Currently, there is no definite treatment for IRDs. However, recent advances have been reported in the field of gene therapy for RP, Leber's congenital amaurosis, and Stargardt disease.^[11]^ If gene therapy may be speculated to repair the defect in gene function, healthy choriocapillaries are necessary to provide enough blood flow to compensate for the normal metabolic needs of the outer retina.

Enhanced depth imaging (EDI) by spectral domain optical coherence tomography (SD-OCT), is a technique used to visualize the detailed structure of the choroidal tissue from the nasal region adjacent to the optic nerve to the subfoveal choroid and the temporal region of the choroid and can be used to measure the choroidal thickness.^[12]^ Measurement of the choroidal thickness can be informative in determining the pathophysiology and natural course of IRDs.^[13,14,15]^

Some studies have reported a significant reduction in the choroidal thickness in RP,^[4,15,16]^ Stargardt disease,^[17]^ and cone dystrophy^[18]^ when compared with healthy controls. However, no difference was observed in other studies.^[5,19]^ Due to this discrepancy, the aim of the present study was to measure the choroidal thickness in a large Iranian cohort with RP, Stargardt disease, cone-rod dystrophy, and Usher syndrome and to compare it with age-matched healthy subjects using EDI-optical coherence tomography (EDI-OCT). The study also aimed to evaluate the relationship of choroidal thickness with the best-corrected visual acuity (BCVA), central macular thickness (CMT), and the duration of ocular symptoms.

##  METHODS

In this comparative study, 503 eyes of 253 patients diagnosed with IRDs including RP (264 eyes of 133 patients), Stargardt disease (76 eyes of 38 patients), cone-rod dystrophy (109 of 55 patients), and Usher syndrome (54 eyes of 27 patients) were included. For comparison, 109 normal eyes of 56 healthy subjects were included as controls. Healthy controls were matched based on patients' age and sex. Data were extracted from the Iranian National Registry of IRDs (IRDRegⓇ). Patients were recalled for additional examinations and imaging according to the standard protocol. This study was conducted at Labbafinejad Medical Center, Tehran, Iran, from January 2016 to August 2018.

The Ethics Committee of the Ophthalmic Research Center, Research Institute for Ophthalmology and Vision Science, Shahid Beheshti University of Medical Sciences, Tehran, Iran approved this study and all procedures were in compliance with the tenets of the Declaration of Helsinki. Written informed consent was obtained from all subjects for diagnostic procedures and choroidal imaging.

### Visual and Ocular Examinations

Initially, all patients were interviewed to identify the age of disease manifestations and the common signs and symptoms including photophobia, color vision deficiency, nyctalopia, nystagmus, restricted visual field, and previous general and ocular conditions. All subjects underwent complete ophthalmic examination including BCVA assessment, color vision testing using Ishihara Pseudoisochromatic plates, slit-lamp biomicroscopy, measurement of the intraocular pressure using the Goldmann applanation tonometer, and dilated fundus examination using a +78D lens. In addition, visual field testing was performed with Humphrey visual field (Carl Zeiss Meditec Inc., Dublin, CA, USA) using 30-2 Swedish Interactive Threshold Algorithm standard method. Additionally, SD-OCT scanning was performed and choroidal thickness was also measured using the EDI-OCT scan (Spectralis, Heidelberg Engineering, Heidelberg, Germany). Fundus photographs were obtained by a digital stereoscopic camera (Visucam Pro NM, Carl Zeiss Meditec AG, Germany). Infrared imaging, fundus autofluorescence, and fluorescein angiography (Heidelberg Engineering GmbH, Heidelberg, Germany) were also performed. In addition, electrophysiological examinations including electroretinography (ERG) and/or electro-oculography (RETIport 21 system, version 7/03, Roland Consult, Osaka, Japan) were conducted to confirm the clinical diagnosis. Based on fundus examination, macular involvement was defined as the presence of any kind of macular abnormality from reduced foveal reflex to bull's eye pattern or beaten bronze appearance.

### Inclusion and Exclusion Criteria

Patients with syndromic RP, IRD cases having optic atrophy due to other etiologies, visually significant cataract or other media opacities, high refractive errors (myopia ≥ 5.00 D, hyperopia ≥ 3.00 D, and astigmatism ≥ 3.00 D), nystagmus or wandering gaze, poor image quality, and any other associated retinal pathologies were excluded. Patients with cystoid macular edema were also excluded from the analysis. We also excluded patients with systemic diseases affecting the choroidal thickness such as systemic hypertension, diabetes, and renal failure. The control group included sex- and age-matched healthy subjects with no ocular and systemic diseases and without high refractive errors. None of the controls had a positive family history of IRDs.

### Final Diagnosis

The Final diagnosis of retinal dystrophy was obtained based on clinical examinations, retinal multimodal imaging, and psychophysical tests such as ERG, color vision, and visual fields. Additionally, retinal dystrophy was confirmed by genetic findings in 98 patients (19.5%). Cross-validation of patients' response with clinical records was performed to increase the data validity.

### Choroidal Thickness Measurement 

**Figure 1:**
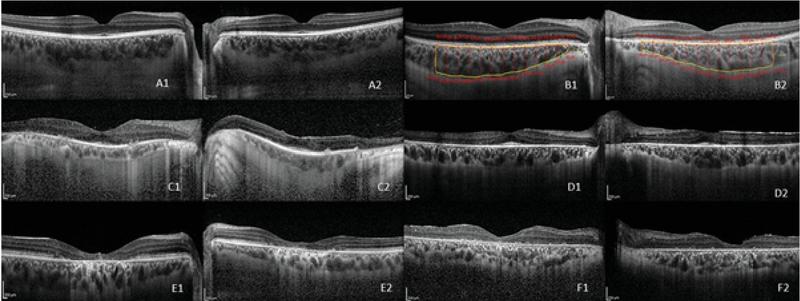
Representative enhanced depth imaging (EDI) of four different inherited retinal dystrophies and of a healthy control. A1: Right eye of a healthy control, A2: left eye of a healthy control B1, B2: Examples of choroidal thickness measurement manually and automatically in the right and the left eyes, respectively, of a healthy subject C1, C2: EDI of the right and the left eyes, respectively, of a patient with retinitis pigmentosa D1, D2: EDI of the right and the left eyes, respectively, of a patient with Usher syndrome E1, E2: EDI of the right and the left eyes, respectively, of a patient with Stargardt disease F1, F2: EDI of the right and the left eyes, respectively, of a patient with cone-rod dystrophy

Each patient underwent an EDI-OCT scan after dilation of the pupil by 1% tropicamide eye drop (Figure 1, A1 to F2). EDI-OCT automatically sets the choroid closer to the zero-delay line and thus, theoretically provides better visualization of the choroidoscleral interface. One horizontal 9mm high-quality line scan through the fovea was obtained for each eye. The line scan was saved for analysis after averaging of 100 frames. Choroidal measurements were performed using MATLAB 2016b program (MathWorks, Natick, MA, USA) at 13 points subfoveally and in nasal and temporal directions with the interval of 500 µm in a length of 6000 µm (Figure 1, B1 and B2).

**Figure 2:**
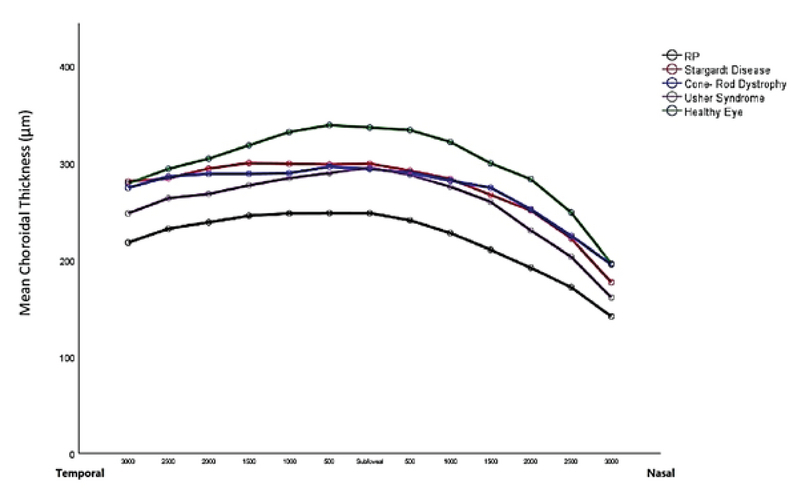
Mean choroidal thickness in the subfoveal area and at six different spots along the nasal and the temporal directions in different types of inherited retinal dystrophies. RP, retinitis pigmentosa

**Figure 3:**
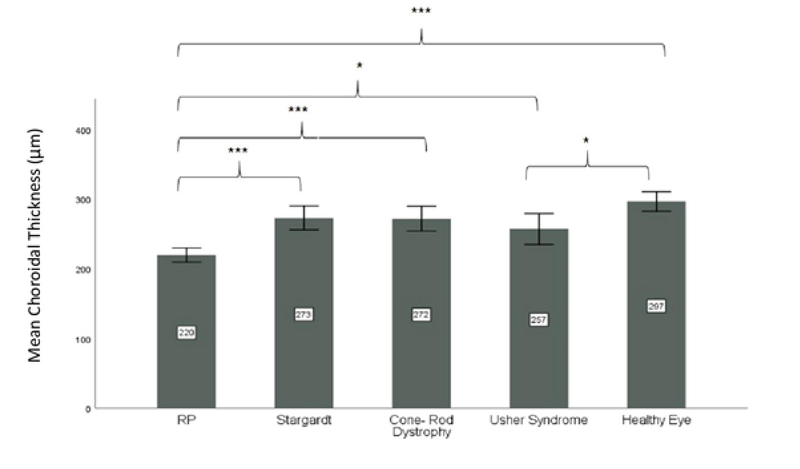
Mean total choroidal thickness (at 13 spots of measurement) in different types of inherited retinal dystrophies. RP, retinitis pigmentosa *Significant *P*-values between 0.01 and 0.05; ***Significant *P*-values < 0.001

**Figure 4:**
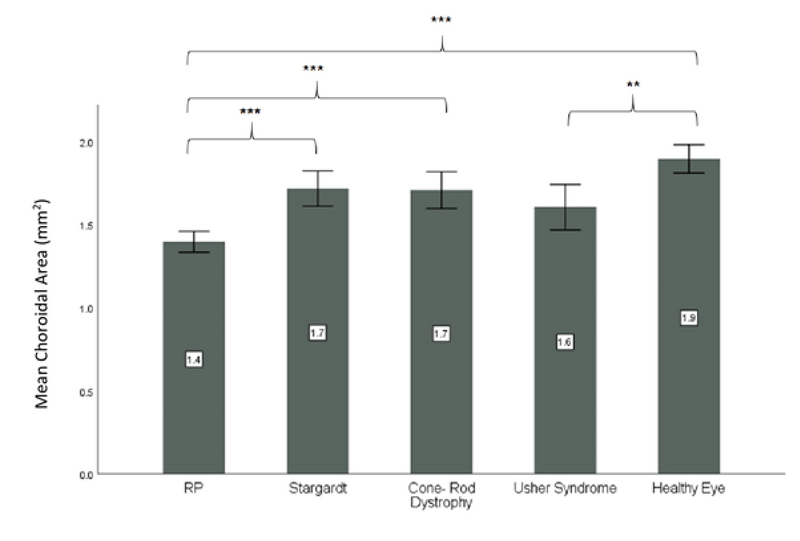
Mean choroidal area in different types of inherited retinal dystrophies RP, retinitis pigmentosa **Significant *P*-values between 0.01 and 0.001 ***Significant *P*-values < 0.001

All choroidal measurements were done by an ophthalmologist familiar with the MATLAB program. All measurements were performed from the outer portion of the hyper-reflective line corresponding to the RPE cells-Bruch's membrane complex as the inner border to the hypo-reflective line corresponding to the sclerochoroidal interface as the outer border. Subsequently, the program automatically measured choroidal thickness in the specified spots according to the spaces defined in the preceding step, measured the area of choroid in the specified region, and provided the information in an excel file. The aforementioned application was designed to work as follows.

1. An EDI-OCT image was displayed in a window after loading the image into the application. The coordinates of all pixels and their intensities were shown in the image.

2. The program allowed the physicians to manually select the center of macula and the choroidal region on the EDI-OCT images.

3. Based on the specified center and the area, six points with 500 μm intervals on each side of the center (13 points including the center) were automatically marked and the choroidal thickness was calculated at these points.

4. The program also provided charts related to changes in the choroidal thickness and other additional information including the area of the choroidal region.

All EDI-OCT images from the patients and the controls were obtained from 2:00 to 6:00 pm to reduce the possible effect of diurnal variation on choroidal thickness. Finally, two other board-certified retina specialists independently rechecked the measurements to avoid disagreements (*Cronbach's α* = 0.794). In a few images with disagreement, the RPE-Bruch's membrane complex and the sclerochoroidal interface were rechecked and the measurements were repeated to be confirmed by the two retina specialists.

### Statistical Analysis

Data were presented as mean and standard deviation, median and range, and frequency and percentage. To compare the subject characteristics between the IRD and the control groups, we used Dunnet's correction for multiple comparisons. To determine the possible correlation of the patients' characteristics or the IRD characteristics with the choroidal thickness and the choroidal area, generalized estimating equation analysis was used. All statistical analyses were performed using IBM SPSS Statistics for Windows, version 25.0. (IBM Corp., Armonk, NY, USA). All tests were two-sided and a *P*-values < 0.05 were considered statistically significant.

##  RESULTS

In this comparative study, 503 eyes with a confirmed diagnosis of IRD including three types of diffuse photoreceptor dystrophies (RP, Usher syndrome, and cone-rod dystrophy) and one macular dystrophy (Stargardt disease) were included. The age and gender distribution of the patients and controls are presented in Table 1.

We observed that for most of the cases, the duration since first recognition of the disease was 10 to 20 years (30.5%, *P *= 0.04). The distribution of the IRD duration in 10-year periods was not significantly different among the types of IRDs.

Table 2 summarizes the clinical characteristics of the study subjects. Comparison of central vision among different types of IRDs showed that the mean BCVA of patients with cone-rod dystrophy was significantly lower than that of patients with Stargardt disease (*P *= 0.009) and Usher syndrome (*P* < 0.001).

The mean CMT values of patients with Stargardt disease and cone-rod dystrophy were significantly lower than those of patients from other groups (*P* < 0.001).

Figure 2 shows the linear comparison of mean choroidal thickness at each of the 13 different spots in different IRDs and the mean choroidal thickness in the control group. The mean subfoveal choroidal thickness in healthy controls (336.79 ± 82.75 µm) was greater than that in RP patients (*P* < 0.001), but did not differ significantly from mean subfoveal choroidal thickness in other IRDs including Usher syndrome, cone-rod dystrophy, and Stargardt disease.

The mean total choroidal thickness (mean choroidal thickness at 13 points) in eyes with RP (*P* < 0.001) and Usher syndrome (*P* = 0.042) was significantly lower than that in healthy eyes. There was no significant difference in mean total choroidal thickness between patients with Stargardt disease and cone-rod dystrophy vs. healthy controls (*P* = NS). The mean choroidal thickness in RP patients was less than that in patients with Stargardt disease (*P* < 0.001), cone-rod dystrophy (*P* < 0.001), and Usher syndrome (*P* = 0.028) (Figure 3).

**Table 1:** Basic characteristics of the study subjects


		**Groups**					
							
**Parameters**	**Levels**	**Total (** ***n*** ** = 612)**	**RP (** ***n*** ** = 264)**	**Stargardt (***n***** = 76)****	**Cone- Rod Dystrophy (***n***** = 109)****	**Usher Syndrome (***n***** = 54)****	**Control (***n***** = 109)****	**** ***P*** **-value**
Age (years)	Mean ± SD	35.75 ± 12.81	40.21 ± 13.03	29.47 ± 8.82	32.33 ± 13.52	35.04 ± 11.01	32.78 ± 11.07	< 0.075*
	Median (range)	34 (5 to 72)	39 (8 to 72)	30 (9 to 45)	32 (8 to 64)	36 (5 to 53)	31 (11 to 65)	
Sex (%)	Male	149 (48.4%)	69 (51.9%)	17 (44.7%)	30 (54.5%)	10 (37.0%)	23 (41.8%)	0.41*
	Female	159 (51.6%)	64 (48.1%)	21 (55.3%)	25 (45.5%)	17 (63.0%)	32 (58.2%)	
Incidence Age (years)	Mean ± SD	18.87 ± 13.09	20.65 ± 14.16	17.05 ± 9.46	17.08 ± 12.89	16.38 ± 11.85	____	0.171*
	Median (range)	17 (0 to 63)	18 (0 to 63)	15 (6 to 40)	16 (0 to 46)	14 (1 to 40)	
Duration of Disease (years)	Mean ± SD	18.96 ± 13.21	22.22 ± 14.57	12.42 ± 8.8	15.83 ± 11.34	18.73 ± 10.08	_____	< 0.001*
	Median (range)	16.5 (0 to 61)	21 (0 to 61)	11 (1 to 35)	14 (0 to 45)	16.5 (4 to 40)	
Duration of Disease	0 – 10	69 (28.0%)	29 (22.7%)	17 (44.7%)	17 (32.7%)	4 (15.4%)	_____	0.04†
	10 – 20	75 (30.5%)	34 (26.6%)	12 (31.6%)	19 (36.5%)	10 (38.5%)	
	20 – 30	49 (19.9%)	27 (21.1%)	7 (18.4%)	8 (15.4%)	7 (26.9%)	
	30 – 40	31 (12.6%)	18 (14.1%)	2 (5.3%)	7 (13.5%)	4 (15.4%)	
	40 – 50	17 (6.9%)	15 (11.7%)	0 (0.0%)	1 (1.9%)	1 (3.8%)	
	50 – 60	5 (2.0%)	5 (3.9%)	0 (0.0%)	0 (0.0%)	0 (0.0%)	
RP, retinitis pigmentosa; SD, standard deviation; n, number *P-value is based on ANOVA (in all the above analysis, multiple comparison correction have been done with Bonferroni method) †Based on Chi-square test								

**Table 2:** Clinical characteristics of the study subjects


		**Groups**					
							
**Parameters**	**Level**	**Total (** ***n*** ** = 612)**	**RP (** ***n*** ** = 264)**	**Stargardt (** ***n*** ** = 76)**	**Cone- Rod Dystrophy (***n***** = 109)****	**Usher Syndrome (** ***n*** ** = 54)**	**Control (** ***n*** ** = 109)**	**** ***P*** **-value**
BCVA (LogMAR)	Mean ± SD	1.04 ± 0.91	1.19 ± 0.98	1.02 ± 0.47	1.43 ± 0.87	0.84 ± 0.69	0 ± 0	< 0.001
	Median (range)	0.8 (0 to 2.79)	0.8 (0 to 2.79)	0.9 (0.1 to 2.31)	1.31 (0 to 2.7)	0.74 (0.1 to 2.7)	0 (0 to 0)	
SE (D)	Mean ± SD	–1.0 ± 1.78	–0.88 ± 1.79	–1.54 ± 1.33	–0.88 ± 2.07	–1.67 ± 1.94	–0.33 ± 0.94	< 0.001
	Median (range)	–0.75 (–6.63 to 2.75)	–0.5 (–5.75 to 2.75)	–1.25 (–5.25 to 0.75)	–0.31 (–6.63 to 2.63)	–1.5 (–5.75 to 1.5)	–0.38 (–2 to 2)	
CMT (µm)	Mean ± SD	215.99 ± 72.1	230.66 ± 68.81	165.54 ± 65.04	186.03 ± 48.59	244.88 ± 99.87	258.72 ± 15.71	< 0.001
	Median (range)	213 (80 to 690)	222 (95 to 674)	141 (80 to 378)	186.5 (88 to 317)	229.5 (90 to 690)	259.5 (220 to 285)	
Color Vision	Normal	164 (28.1%)	40 (16.1%)	6 (8.3%)	2 (1.9%)	7 (14.6%)	109 (100.0%)	< 0.001
	CVD	213 (36.5%)	71 (28.6%)	53 (73.6%)	62 (57.9%)	27 (56.3%)	0 (0.0%)	
	N/A	207 (35.4%)	137 (55.2%)	13 (18.1%)	43 (40.2%)	14 (29.2%)	0 (0.0%)	
Cataract Type (%)	No	376 (62.0%)	101 (38.5%)	69 (95.8%)	80 (73.4%)	17 (31.5%)	109 (100.0%)	
	CC	0 (0.0%)	0 (0.0%)	0 (0.0%)	0 (0.0%)	0 (0.0%)	0 (0.0%)	< 0.001
	NS	44 (7.3%)	29 (11.1%)	0 (0.0%)	11 (10.1%)	4 (7.4%)	0 (0.0%)	
	PSC	169 (27.9%)	120 (45.8%)	3 (4.2%)	16 (14.7%)	30 (55.6%)	0 (0.0%)	
	PCIOL	14 (2.3%)	12 (4.6%)	0 (0.0%)	2 (1.8%)	0 (0.0%)	0 (0.0%)	
	Posterior Polar	3 (0.5%)	0 (0.0%)	0 (0.0%)	0 (0.0%)	3 (5.6%)	0 (0.0%)	
Optic Atrophy (%)	No	46 (9.1%)	10 (3.8%)	22 (28.9%)	14 (12.8%)	0 (0.0%)	0 (0.0%)	< 0.001
	Yes	457 (90.9%)	254 (96.2%)	54 (71.1%)	95 (87.2%)	54 (100.0%)	0 (0.0%)	
Macular Involvement (%)	No	122 (19.9%)	11 (4.2%)	0 (0.0%)	2 (1.8%)	0 (0.0%)	109 (100.0%)	< 0.001
	Yes	490 (80.1%)	253 (95.8%)	76 (100.0%)	107 (98.2%)	54 (100.0%)	0 (0.0%)	
RP, retinitis pigmentosa; BCVA, best corrected visual acuity; LogMAR, logarithm minimum angle of resolution; SE, spherical equivalent; D, diopter; CMT, central macular thickness; µm, micrometer; CVD, color vision defect; N/A, not applicable; CC, cortical cataract; NS, nuclear sclerotic; PSC, posterior subcapsular; SD, standard deviation; n, number *These parameters are presented based on monocular findings								

The mean total choroidal area in different types of IRD and in healthy controls is illustrated in Figure 4. The mean total choroidal area in healthy subjects (1.9 ± 0.4 µm${}^{2}$) was significantly more than that in RP (1.4 ± 0.5 µm${}^{2}$, *P* < 0.001) and Usher syndrome (1.6 ± 0.5 µm${}^{2}$, *P* = 0.006). Among different IRDs, the mean total choroidal area was less in RP patients than in patients with Stargardt disease (*P* < 0.001) and cone-rod dystrophy (*P* < 0.001).

Additionally, the subfoveal choroidal thickness was inversely correlated with BCVA (*r* = -0.264, *P* < 0.001) and the duration of ocular symptoms (*r* = -0.341, P < 0.001) in all IRDs. However, no statistically significant correlation was observed between the subfoveal choroidal thickness and CMT (*r* = -0.24, *P* = 0.576) in all IRDs.

##  DISCUSSION

In the present comparative study, choroidal thickness was measured in a large group of patients with different types of IRDs including RP, Stargardt disease, Usher syndrome, and cone-rod dystrophy. We included age- and sex-matched controls for comparison.

The mean choroidal thickness measured at 13 different spots in the nasal and in the temporal direction was significantly lower in patients with RP and Usher syndrome when compared with healthy controls and patients with Statgardt disease and cone-rod dystrophy. Other studies have also reported similar findings.^[4,5,13,16]^ However, no significant reduction in choroidal thickness was reported in RP patients in the study by Chhablani et al.^[19]^ This discrepancy could be attributed to the different characteristics of the study population including better BCVA (0.99 ± 0.94 LogMAR) and younger age (31.09 ± 13.40 years) than the BCVA (1.19 ± 0.98 LogMAR) and age (40.21 ± 13.03 years) of patients in our study. High number of patients with RP (*n* = 264) was a strength of the present study. The number of RP patients was higher than those included in previous studies.^[4,13,16,19]^ Sodi et al reported findings consistent with our results with no difference in mean choroidal thickness between healthy controls and patients with Stargardt disease.^[5]^ They described a statistically significant correlation between lower subfoveal choroidal thickness and longer duration of ocular symptoms; this finding was also observed in the current study.^[5]^

To the best of our knowledge, this is the first study that compared the choroidal thickness among different types of IRDs. Our investigation showed that the lowest choroidal thickness was observed in patients with RP. This finding could be explained by the possible trophic role of RPE cells, which support proper choroidal function through secretion of growth factors. Conversely, the reduction in oxygen demand of the degenerating photoreceptors will subsequently result in reduced blood flow to the retina and the choroid, which is called primary vascular dysregulation in RP patients.^[20,21]^ Additionally, RP has been associated with an imbalance in oxidant/antioxidant status and subclinical inflammatory processes that may stimulate the excessive production of endothelin-1 (ET-1).^[16,19]^ Therefore, retinal degeneration and choroidal thinning in patients with RP seem to interact with each other.

The difference in the amount of choroidal thinning between RP and other IRDs can be explained by the pathogenesis of RP.^[16]^ Most of the mutations in RP are attributed to gene coding of proteins involved in the vision cycle at the level of photoreceptors and RPE cells. These mutations cause apoptosis and degeneration of photoreceptors and subsequent outer retinal thinning.^[16,22]^ Photoreceptor atrophy results in reduced oxygen demand and blood flow. Furthermore, trophic role of RPE cells through secretion of growth factors such as vascular endothelial growth factor^[23]^ that support proper choroidal function is diminished. Thus, choroidal thinning can be explained by reduced blood flow and RPE cell degeneration.

Yoshida et al observed cell, flare, and inflammatory cytokines in aqueous humor and vitreous fluid of patients with RP.^[24]^ Confirmation of reduced level of antioxidants in this group of patients by Martinez et al is suggestive of the oxidative stress in RP.^[25]^ Subclinical ocular inflammatory process and hypoxic-oxidative stress elicit increased ocular and plasma levels of ET-1.^[16,22]^

ET-1 is the most powerful endogenous vasoconstrictor of small and large vessels. In the eye, its synthesis and secretion is performed by different tissues such as cornea, uveal tissue, retinal microvascular pericytes, RPE cells, and optic nerve. Increased levels of ET-1 and vasoconstriction effect result in vascular dysgenesis and impaired ocular blood flow. This vicious cycle contributes to the amplification of the inflammatory response, altered intraocular perfusion, relative ischemia, and consequent degeneration of outer retinal layer and choroidal thinning.^[22]^

In the present study, a negative correlation was observed between the subfoveal choroidal thickness and BCVA and between subfoveal choroidal thickness and disease duration of the IRDs. However, no correlation was observed between CMT and choroidal thickness. Some studies have reported a correlation between choroidal thickness and BCVA and between choroidal thickness and the duration of ocular symptoms in IRDs including RP and Stargardt disease.^[4][5,19]^ However, other studies did not find such correlations.^[10,13,16,18]^ This difference may be due to a higher number of subjects and a longer duration of ocular symptoms in the present study. Ayton et al^[4]^ reported that choroid becomes thinner with increasing duration of RP symptoms, which is consistent with the findings of the present study. However, Sodi et al^[16]^ claimed that age might have more effect on choroidal thickness than the duration of RP itself. They suggested that this lack of association might be due to a very strict criterion for the definition of age at the onset of the disease. In the present study, additional age-adjusted analysis confirmed that age could not be a confounding variable and the findings may be directly related to the IRD entity.

Previous studies have found a significant correlation between CMT and choroidal thickness in Stargardt disease^[5]^ and no relationship between them in RP patients.^[13][16,19]^ In the present study with a larger sample size, no correlation was observed between CMT and subfoveal choroidal thickness in RP and in Stargardt disease. As previously stated, patients with cystoid macular edema were not included in the statistical analysis.

Total choroidal area in the eyes of healthy subjects was significantly higher than that in eyes of patients with RP and Usher syndrome. The choroid was generally thinner in RP patients when compared with patients having Stargardt disease and cone-rod dystrophy.

One of the strengths of the present study is the comparison of choroidal thickness among four relatively common types of IRDs including RP, Stargardt disease, Usher syndrome, and cone-rod dystrophy. Additionally, a larger sample size compared to the sample size in previous studies is another merit of this study. The design of the novel software using MATLAB computer programming for automatic measurement of the choroidal parameters minimizes the chance of inter-operator and intra-operator errors. The measurement of the total choroidal area as well as the choroidal thickness at 13 different spots (including the center of the fovea) 6000 µm along the nasal and the temporal directions from the center of the fovea is another merit of the present study.

The present study has some limitations including the lack of accessibility to the genetic data for all study subjects. Another limitation of this study was relying on patients' self-report for identification of the age of disease onset. Of course, cross-validation of patients' response with clinical records was performed to increase the data validity.

In conclusion, with the same duration of ocular symptoms, generalized choroidal thinning was observed in RP and Usher syndrome, but not in Stargardt disease and cone-rod dystrophy. Therefore, different pathophysiologic and blood flow mechanisms may be implicated in each IRD, which demands further cohort studies with special consideration of choroidal blood flow as a potential therapeutic target.

##  Financial Support and Sponsorship

The study data were extracted from the Iranian National Registry of IRDs (IRDRegⓇ), which is financially supported by the Deputy of Research and Technology of the Iranian Ministry of Health and Medical Education, as well as Shahid Beheshti University of Medical Sciences, Tehran, Iran.

##  Conflicts of Interest

There are no conflicts of interest.
